# Editorial: Protein recognition and associated diseases

**DOI:** 10.3389/fbinf.2023.1215141

**Published:** 2023-05-22

**Authors:** M. Michael Gromiha, Petras Kundrotas, Marcelo Adrian Marti, Česlovas Venclovas, Minghui Li

**Affiliations:** ^1^ Department of Biotechnology, Bhupat and Jyoti Mehta School of Biosciences, Indian Institute of Technology Madras, Chennai, Tamil Nadu, India; ^2^ Center for Computational Biology, The University of Kansas, Lawrence, KS, United States; ^3^ Departamento de Química Biológica, Facultad de Ciencias Exactas y Naturales, Universidad de Buenos Aires (FCEyN-UBA) e Instituto de Química Biológica de la Facultad de Ciencias Exactas y Naturales (IQUIBICEN) CONICET, Pabellòn 2 de Ciudad Universitaria, Buenos Aires, Argentina; ^4^ Institute of Biotechnology, Life Sciences Center, Vilnius University, Vilnius, Lithuania; ^5^ Center for Systems Biology, Department of Bioinformatics, School of Biology and Basic Medical Sciences, Soochow University, Suzhou, China

**Keywords:** protein-protein interactions, binding affinity, machine learning, protein-protein interaction networks, phylogenetic profiles

Protein-protein interactions are essential for many biological functions in all living organisms including cell signaling, molecular switching, transporters, receptors, and immunity. For the past few decades, tremendous advancements have been made in order to understand the recognition mechanism of protein-protein complex formation, reconstruct protein-protein interaction networks of an entire organism, and/or complete biochemical pathways. These efforts are mainly focussed on the identification of interacting proteins, prediction of binding site residues at their interface, evolutionary conservation of protein-protein complexes, prediction of protein-protein complex structures by docking, predicting the binding affinity of protein-protein complexes, and assessing the mutational effects on strength of binding and diseases (Gromiha, 2020). Recently, AlphaFold (Jumper et al., 2021) and its descendants (e.g., AlphaFoldMultimer, Evans et al., 2021), have demonstrated spectacular success in predicting structures of individual proteins and their complexes. Nevertheless, a significant number of cases and questions are still evading solutions and answers. This Research Topic on “*Protein Recognition and Associated Diseases*” addresses the recent advances in computational methodologies for the analysis and identification of important residues for binding, scoring, and ranking of structural models of protein-protein complexes, protein-protein interaction networks, and their applications in life sciences and human health.

The opening article by Brysbaert and Lensink analyzes the performance of several centrality measures for identifying major interacting residues involved in protein-protein binding using binding affinity data of interface mutations. Johansson-Åkhe et al. propose a machine learning-based method for scoring and ranking peptide-protein complexes. It encodes the structure of the complex as a graph with evolutionary and sequence features as nodes and physical pairwise interactions as edges. Su et al. integrate protein-protein interaction networks and gene expression profiles for detecting pancreatic adenocarcinoma candidate genes. Karan et al. report the development of four genomic information-based prediction methods, namely, 1) interolog, 2) domain, 3) gene ontology, and 4) phylogenetic for identifying the interaction between *Oryza sativa* and *Magnaporthe grisea* in a whole-genome scale.

In essence, this Research Topic covers the exciting developments in the area of protein-protein interactions both at fundamental and application levels. It will be a valuable resource for computational biologists, biochemists, biophysicists, bioinformaticians, and researchers working in the field of protein-protein interactions, and for those working on the biological role of protein-protein interaction networks and their relation to disease.

We would like to thank all the authors for their outstanding contributions. The guest editors thank the Editorial Assistant Dr. Sara Gomez Cabellos, Commissioning Specialist, and Dr. Rahila Esposito, Commissioning Manager for their help and support in successfully completing the Research Topic.
